# Feasibility of Mindfulness for Burn Survivors and Parents of Children with Burns

**DOI:** 10.3390/ebj4020020

**Published:** 2023-05-24

**Authors:** Eleni Papamikrouli, Marianne B. Kool, Carine van Schie, Nancy E. E. Van Loey

**Affiliations:** 1 Dutch Burns Foundation, 1940EA Beverwijk, The Netherlands; 2 Netherlands Cancer Institute, 1066CX Amsterdam, The Netherlands; 3 Association of Dutch Burn Centers, Maasstad Hospital, 3079DZ Rotterdam, The Netherlands; 4 Dept Clinical Psychology, Utrecht University, 3584CS Utrecht, The Netherlands

**Keywords:** burns, mindfulness, group intervention, parents, MBSR

## Abstract

Burn survivors, spouses, and parents of children with burns may experience psychological distress for a prolonged period. Mindfulness-Based Stress Reduction (MBSR) is an intervention that can improve psychological well-being. This study aimed to examine the effectiveness of an MBSR group intervention in a convenience sample. An MBSR group intervention was conducted for burn survivors (*n* = 8) and parents of children with burns (*n* = 9), each comprising eight sessions. The participants completed the Beck Depression Inventory-II-NL, PTSS Checklist DSM-5, Five Facet Mindfulness Questionnaire-Short Form, Self-Compassion Scale-Short form, and evaluation questions at baseline, immediately after, and three months post-intervention. All participants completed the intervention. The intervention was rated very useful (M = 8.8), and the participants were very satisfied (M = 8.8). The highest effect was observed in the parents’ group on mindfulness skills and self-compassion. For both groups, there was an increase in personal goal scores immediately after the intervention. Qualitative data show that the participants in both groups experienced more inner peace, more awareness of thoughts and emotions, and more self-compassion. This exploratory study suggests that a mindfulness intervention is feasible and can be effective in improving mindfulness skills and self-compassion, particularly in parents of children with burns.

## 1. Introduction

A burn injury can be a traumatic and painful experience. Unsurprisingly, psychological problems, such as posttraumatic stress symptoms, anxiety, and depressive symptoms, can interfere with recovery, increasing pain and itch, disrupting sleep patterns, and affecting interpersonal relationships [1,2,3,4]. Several studies show that psychological and social challenges may persist for years after the injury [5,6,7,8]. Parents of children with burns may also experience psychological distress in the aftermath of the burn event. Posttraumatic stress symptoms, depressive symptoms, and guilt feelings are among the most frequently reported problems, resulting in chronic problems in a subsample of parents [9,10,11,12,13]. However, good family functioning is important to improve the outcomes for children who have sustained a burn injury [14,15], and research shows that parents’ mental health is also important for the child’s general health, providing a rationale for supporting parents [16]. Furthermore, spouses may also be affected by the burn event, resulting in symptoms of depression and posttraumatic stress [17,18]. Given the potential psychological impact of a burn event on burn survivors, spouses, and parents of children with burns, it is important to investigate psychological interventions that may diminish the level of distress.

Mindfulness interventions are increasingly implemented to improve the symptoms of a variety of psychological problems [19]. Specifically, Mindfulness-Based Stressed Reduction (MSBR) is a type of meditation practice that is directed at promoting a controlled, nonjudgmental, and non-evaluative awareness of moment-to-moment experiences [20]. MBSR aims to reduce distress and improve a person’s wellbeing through a series of practices, such as attentively scanning the body, awareness of the breath, mindful walking, and yoga. Recent meta-analyses showed that MBSR can effectively reduce stress and symptoms of anxiety and depression and enhance the quality of life in healthy individuals [21,22]. The efficacy of MBSR has also been investigated in a variety of clinical populations suffering from chronic conditions and diseases, for example, chronic pain [23] and cancer [24]. Empirical evidence supporting the relationship between psychological wellbeing and mindfulness in burn populations is scarce, but a few available studies showed that lower levels of mindfulness (e.g., mindfully describing, which involves finding the words to describe one’s feelings) have been associated with higher levels of appearance anxiety [25]. Other studies have indicated that burn-related quality of life was significantly and positively correlated with mindfulness, and mindfulness was associated with less psychological distress [26,27].

It is worth noting that MBSR is an accessible intervention because it is aimed at learning mindfulness skills together with others, for example, peers, and it is applicable for clinical as well as non-clinical populations. This differentiates MBSR from, for example, Cognitive Behavior Therapy, which is a common psychological intervention for persons with symptoms within the clinical range [28]. The low threshold accessibility makes MBSR suitable for virtually everyone. Moreover, peer support has been shown to facilitate learning in MBSR programs [29]. In burn populations, several studies have highlighted the positive effects of peer support, including enhanced recovery and empowerment, and connection with peers on an emotional level [30,31,32]. Therefore, it was assumed that a group intervention including peers may strengthen the effects of MBSR.

In this exploratory mixed-method study, we investigated the feasibility and potential effectiveness of a mindfulness intervention in a group of burn survivors and a group of parents of children with burns who experienced mild to moderate distress.

## 2. Materials and Methods

Participants in this study were recruited after a one-hour mindfulness workshop at the annual national day for burn survivors, parents of children with burns, and other persons who act as significant others. Potential participants were also recruited through calls announced on social media by the Dutch Burns Foundation. The inclusion criteria for participation were >6 months post-burn, sufficient Dutch proficiency, and having the intention and opportunity to attend at least six sessions of the MBSR program and complete the homework. The exclusion criteria were severe levels of depressive and posttraumatic stress symptoms, i.e., scoring above the established cut-off scores, not able to attend three or more sessions, and having participated in mindfulness training (i.e., ≥3 training sessions) less than 5 years ago.

This study was conducted in accordance with the principles of the Declaration of Helsinki (revision, Fortaleza, Brazil, 2013). The Medical Ethical Committee of North Holland, Netherlands, reviewed and approved the study (NL56533.094.16). All participants provided written informed consent. The study consisted of a pre-intervention assessment, a post-intervention assessment (i.e., 1 week post-intervention), and a follow-up assessment (i.e., 3 months post-intervention). Prior to the start of the MBSR intervention, the participants were interviewed, and they completed questionnaires measuring their depressive and PTSD symptoms, mindfulness skills, self-compassion, personal goals, and demographic characteristics. Immediately after the MBSR intervention (post-intervention test) and 3 months later, they completed all the questionnaires and the additional evaluation questions. At the 3-month follow-up measurement, they were also interviewed by telephone. The eight intervention sessions took place in 2016 in Utrecht, a city located in the center of the country. Travel expenses were reimbursed.

The MBSR intervention followed the program manual that was developed by Kabat-Zinn (1990) [20] at the Stress Reduction Clinic at the Massachusetts Medical Centre provided by two qualified mindfulness trainers. The content of the sessions is described in Table 1. Furthermore, elements of Compassion Training [33] and Breathworks [34] were added to the program. Compassion Training aims to stimulate more compassion towards the self and others. Breathworks is a method that teaches people to cope with pain by “letting go of” frustration and suffering that is associated with pain through breathing techniques and meditation.

Burn survivors and parents of children with burns took part in two separate groups on two different days but had the same trainers. The program consisted of eight sessions of two and a half to three hours and one exercise day of six hours between sessions 6 and 7. The program was delivered within a period of 15 weeks with one-, two-, or three-week intervals. The participants received a handbook summarizing each weekly session, self-help materials, and a meditation CD. They were encouraged to practice daily mindfulness meditation for about an hour and to read the information in the handbook. Examples of practices are: the body scan, attention to breathing, moving meditation, silent practice, and reporting feelings and thoughts in a diary.

Several measures were used. The Checklist for the DSM-5 (PCL-5; Dutch version) [35] is a 20-item self-report instrument indexing the DSM-5 symptom criteria for posttraumatic stress disorder. The self-report rating scale indicates the degree of distress associated with each symptom (ranging from 0 = not at all, to 4 = extremely). Higher scores indicate more severe symptoms. It consists of four subscales: re-experiencing, (five items), persistent avoidance (two items), negative alterations in cognitions and mood (seven items), and increased arousal (six items). A PCL-5 cut-off-point of 38 was used to indicate clinically relevant levels of PTSD. Respondents scoring above this cut-off point were excluded from the study. Cronbach’s alpha ranged between 0.89 and 0.94 in this study.

Beck Depression Inventory-II-NL (BDI-II-NL) [36] is a 21-item self-report instrument that measures depressive symptoms based on the DSM-IV criteria. Higher scores are indicative of more severe symptoms. Items are scored on a 4-point scale ranging from 0 to 3. The cut-off score of 28 indicates severe depression. Respondents scoring above this score were excluded. The BDI-II-NL has demonstrated good internal consistency and test–retest reliability. The Cronbach’s alpha ranged between 0.68 (pre-test) and 0.86 (post-test and follow-up) in this study.

The Dutch Five-Facet Mindfulness Questionnaire—Short Form (FFMQ-SF) [37,38] provides an estimate of the participants’ engagement with the principles of mindfulness. It is a 24-item instrument consisting of five subscales: observing, describing, acting with awareness, non-judgment of inner experience, and non-reactivity to inner experience. The items are rated on a scale ranging from 1 to 5 (never or almost never true to very often or always true), with higher scores indicative of more mindfulness engagement. A total score can be calculated as the sum of all items. The internal consistencies of the five subscales have been reported as moderate to good. The Cronbach’s alpha ranged between 0.63 (pre-test) and 0.82 and 0.84 in this study.

The Self-Compassion Scale (SCS, Dutch version) [39] is a 24-item self-report scale that measures beliefs and attitudes about self-compassion. It consists of six subscales: self-kindness, self-judgment, common humanity, isolation, mindfulness, and over-identification. Items are rated from 1 (almost never) to 5 (almost always) and summed to create subscale total scores, with higher scores indicating a more compassionate self-attitude. The internal consistencies of the subscales have demonstrated adequate internal consistency. The Cronbach’s alpha was 0.92 in this study.

Personal goals were assessed by asking each participant what they hoped to achieve with the MBSR program. The participants could formulate one to five goals. Each goal was reformulated into a self-report rating scale from 0 (never) to 10 (always). For example, the goal “I want more peace of mind” was reformulated into “I experience peace of mind”. Higher scores indicate improvement in the goal.

Satisfaction with the MBSR program was assessed with a self-developed 5-item questionnaire. The items were rated from 0 (not at all) to 10 (very much). How the participants experienced the different aspects of the MBSR program in terms of usefulness was measured with a 10-item self-developed questionnaire. The items were rated from 0 (not useful at all) to 4 (very useful). Furthermore, the participants were asked how pleasant the various aspects of the MBSR were perceived using an 8-item self-developed questionnaire. The items were rated from 0 to 2 (pleasant, neutral, and not pleasant, respectively).

The participants were interviewed by telephone after the intervention was completed to evaluate the intervention and ask about their personal experiences. First, they received information about the questionnaire results and their self-determined goals (improvement or no improvement), and they were asked how they felt about it and to provide more information about what could have caused (no) improvement. Second, they were asked what stimulated or prevented attending the meetings, what their general opinion was about the training, and how they reflected on the training. The answers were coded and categorized by two researchers.

Quantitative analyses were conducted in SPSS (IBM SPSS Statistics for Windows, Version 22.0, Armonk, NY: IBM Corp.). The feasibility of the training was examined by assessing the percentage of eligible participants, attrition and attendance rates, and the participants’ satisfaction (rating scale of 1–10). The attrition and attendance rates were recorded. The effectiveness of the intervention was examined with paired sample *t*-tests. A post-intervention test and the follow-up test were compared with the pre-intervention test. The effect sizes (ES), indicated by Cohen’s d (or standardized mean difference) were calculated and can be interpreted as a small (d = 0.2), medium (d = 0.5), or large (d = 0.8) effect size. Concerning the personal goals, a mean goal score was calculated for each participant and compared with the mean goal score on the post- and follow-up tests.

## 3. Results

### 3.1. Participants

Seventeen participants (seven burn survivors, one spouse, and nine parents) were included in the study. Table 2 shows the characteristics of the participants. Both groups were heterogeneous in terms of years post-burn, varying from 4 to 25 years after the accident for the burn survivors group and from 2 to 34 years after the accident for the parent group.

### 3.2. Feasibility and Acceptability

Of the 28 participants who signed up for the study, three participants were excluded after the intake procedure because they had high levels of depressive and/or posttraumatic stress symptoms. They were advised to discuss their problems with the general practitioner in order to estimate whether individual psychological therapy was indicated. Eight participants failed to attend more than three sessions on the planned dates and were excluded. Seventeen participants (61%) participated in the MBSR intervention. Out of the 17 participants, 8 attended all sessions (47%), 5 were unable to attend 1 session (29%), and 4 could not attend 2 sessions (23%). All participants completed the intervention.

Satisfaction with the MBSR program, assessed immediately after the intervention, is shown in Table 3. The participants in both groups were overall satisfied with the MBSR program, found it useful, and would recommend it to another person with burns or to another parent of a child with burns. The majority of the participants were confident that the effects would last more than 6 months.

**Figure 1:**
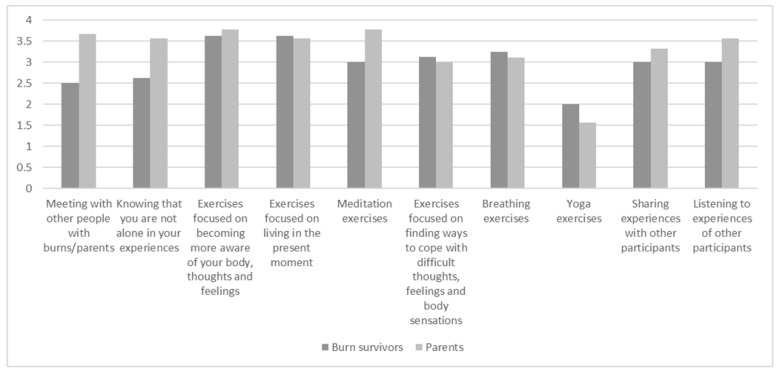
Participants’ ratings of usefulness of the MBSR program components rated from 0 (not useful at all) to 4 (very useful).

Figure 1 shows the participants’ ratings of the usefulness of the various components of the MBSR program. Most of the components were found useful, excluding yoga exercises.

Table 4 presents the agreeability of the intervention aspects (i.e., percentage of participants scoring the various aspects of the program as pleasant), which indicates that the homework and yoga exercises were not found pleasant. Sharing experiences and listening to peers was rated 100% agreeable, indicating that the peer support aspect was highly valued.

Qualitative quotes about peer support: *“The connection that you find in a group of peers is special. A place where you can do something for each other. In addition to practising mindfulness, this is another added value of these group meetings.“*

Qualitative quote about usefulness: *“I have experienced the training as meaningful; it has made me much more aware of my feelings and thoughts. I’m avoiding less and I am going into confrontation with my feelings, no matter how hard that is. I have felt, cried and processed more in recent months than all the years before. I find this very valuable and I am very happy that I did the training.”*

### 3.3. Effectiveness

#### 3.3.1. Depressive and PTSD Symptoms

**Figure 2:**
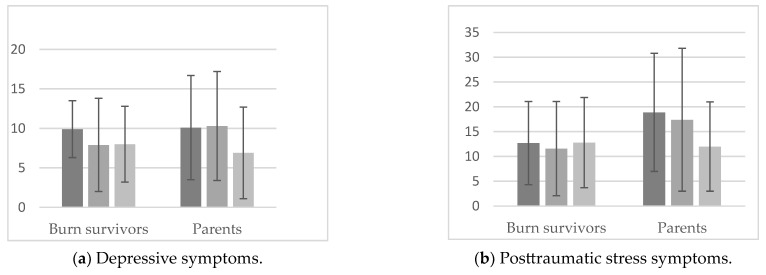

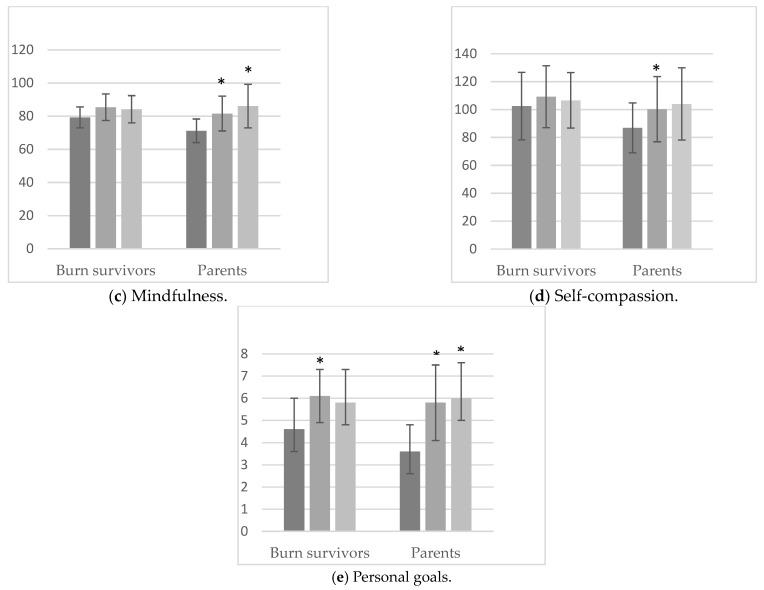
Mean scores and standard deviations for pre-intervention (left bar), post-intervention (middle bar), and follow-up (right bar) test scores for the respective measures for the burn survivor group and parent group. * Indicates statistically significant differences at the 0.05 level compared to the pre-intervention score.

Depressive symptoms decreased in both groups, but this was not statistically significant in either the post-intervention (PI) tests or follow-up (FU) tests. The effect sizes (ES) for the burn survivors were d_PI_ = 0.27 and d_FU_ = 0.33; for parents, they were d_PI_ = 0.02 and d_FU_ = 0.44). The PTSD scores decreased in parents, but not statistically significantly (the ES for burn survivors were d_PI_ = 0.10 and d_FU_ = 0.01; for parents, they were d_PI_ = 0.07 and d_FU_ = 0.45). Figure 2 presents the mean scores.

#### 3.3.2. Mindfulness Skills and Self Compassion

For the burn survivor group, no statistically significant changes were found regarding mindfulness (ES: d_PI_ = 0.59 and d_FU_ = 0.51, indicating a medium effect) and self-compassion scores (ES: d_PI_ = 0.23 and d_FU_ = 0.17, indicating a small effect), as presented in Figure 2 and Table 5. In the parent group, a statistically significant increase in the mindfulness total score between the pre-intervention test and the post-intervention test (t(8) = 2.41, *p* = 0.043, d = 1.02) and between the pre-intervention test and the follow-up test (t(8) = 2.74, *p* = 0.026, d = 0.96, indicating a large ES) was found, as shown in Figure 2. More specifically, “describing”, which refers to one’s ability to label their feelings, thoughts, and experiences with words, and “acting with awareness”, which refers to attending to what is happening in the present, showed a significant improvement post-intervention, as shown in Table 5. Regarding self-compassion, the total score significantly increased in the parent group (t(8) = 2.79, *p* = 0.023, d = 0.93), an effect that was particularly related to an increase in the self-kindness subscale, which increased in the post-intervention test (t(8) = 4.12, *p* = 0.003). This also applied to the common humanity subscale (t(8) = 3.45, *p* = 0.011). The subscale of mindfulness also showed an increase in the post-intervention test (t(8) = 3.2, *p* = 0.015). The subscale of self-kindness showed an increase in the post-intervention test (t(8) = 3.8, *p* = 0.006), and self-judgment increased in the follow-up test (t(8) = 2.45, *p* = 0.040).

Despite the lack of statistically significant results in the burn survivors, they reported that the intervention helped them develop mindfulness skills, including greater awareness and more peace and concentration:

*“My thought process has changed (i.e., after participating in mindfulness intervention). I now think before I explode when something is bothering me or when I am arguing with someone.”*

*“I am much more aware of my thoughts and breathing. I still have a lot of practice to do but I’m getting better and better. [I am able] To stay with myself and to set my own boundaries.”*

*“I have learned to take a better look at myself, my emotions and complaints.”*

*“It has brought me inner peace. As a result, I can concentrate better on what I need to do. I am able to handle my emotions better.”*

The parents reported that the intervention helped them to achieve greater awareness of their thoughts and feelings:

*“I better understand what I think and feel. I have become much more aware of that. This gives me a lot of peace and growing confidence in myself.”*

*“I am much more aware of all sorts of feelings either positive or negative about what is going on in my family and work life right now and the physical reactions to it.”*

*“I have learned thoughts are just spins of the brain that run wild with you. This gave me peace of mind. I see that they are just thoughts.”*

More compassion for the self and others was also named by the parents:

*“I have better learned how to deal with stress, with compassion, and understanding for myself. More patience and love for the little one. More inner peace and a better relationship with my partner.”*

*“I have learned to cherish my feelings of guilt and let them go.”*

#### 3.3.3. Personal Goals

The participants formulated several personal goals. The main themes of the participants’ personal goals were: (1) awareness of thoughts and emotions, e.g., “I am aware of what is happening in the now”; (2) peace/tranquility, e.g., “I experience peace in my mind”; (3) self-confidence, e.g., “I stand firmly on my feet”; (4) compassion for self and others, e.g., “being less hard on myself and other”; and (5) physical wellbeing, e.g., ”I listen to my body.” These goals were rated on a scale ranging from 1 to 10.

Significant improvements in personal goals, also indicated by large effect sizes, were found for both groups. Regarding the burn survivors’ personal goals, an improvement was shown from the pre-intervention test to the post-intervention test (t(7) = −2.81, *p* = 0.026, d = 0.99). Regarding the parents’ personal goals, the scores increased significantly post-intervention (t(8) = -2.71, *p* = 0.027, d = 0.90) and in the long term (follow-up) (t(8) = −3.29, *p* = 0.011, d = 1.10).

## 4. Discussion

This study examined the feasibility and effectiveness of an MBSR intervention in a group of burn survivors and a group of parents of children with burn injuries. In terms of feasibility, the satisfactory recruitment (61% met the inclusion criteria and could attend the majority of the sessions) indicates that mindfulness was an intervention that burn survivors and parents of children with burn injuries were willing to attend, and they showed overall satisfaction with the program. Regarding the effectiveness, mindfulness skills and self-compassion improved for the parents of children with burn injuries after the intervention. The personal goal scores increased for both groups immediately after the intervention, and they reported more inner peace, more awareness of thoughts and emotions, and more self-compassion. However, this study also shows that it was challenging to recruit sufficient participants as only 28 people showed interest. Perhaps a shorter and tailored intervention including only useful exercises and leaving out less relevant exercises can decrease the number of sessions and may increase recruitment rates. Furthermore, face-to-face MBSR meetings could be combined with online meetings to overcome the barrier of traveling distance.

The participants in both groups found the intervention useful and agreeable, and overall, they were satisfied with the program. The perception of the participants that the intervention was useful was also mirrored in the qualitative part of the study. The personal goals, including awareness, more peace and tranquility, self-confidence, compassion for one’s self and others, and physical wellbeing improved significantly in the post-intervention test. For parents, this improvement was also maintained at follow-up, which indicates that the intervention provided the participants with useful tools for the attainment of their personal goals. The findings of the current study add to the growing body of literature suggesting that mindfulness may be a useful complementary intervention to reduce psychological distress and has proven feasibility in a variety of (clinical) populations (e.g., women with breast cancer [40]).

All participants appreciated the intervention as an opportunity to come into contact and share experiences with peers. Practicing mindfulness exercises with people with similar experiences was both appreciated and considered helpful by the participants. This is in accordance with previous research [32,41] suggesting that the “ideal peer support program” includes peers in a relaxed and safe atmosphere in combination with recreational activities. A mindfulness intervention, regarded as a recreational intervention with therapeutic potential, can be a safe and relaxing environment in which burn survivors and parents of children with burns can create bonds, offer each other support, and exchange experiences. This is also in accordance with previous studies that have reported that group-based mindfulness interventions were of added value for cancer patients and parents of children with chronic conditions [42,43]. Furthermore, the findings support the surplus value of peer support in MBSR programs [44].

This study suggests that the effectiveness of the intervention was higher for the parents compared to the burn survivors. Although for both groups there was an improvement in mindfulness skills, the largest effect sizes were observed in the parent group. Particularly, ‘mindfully describing’ and ‘awareness’ improved, which corroborates a prior study on burn survivors [25]. Self-compassion also improved in the parents. A possible explanation for the improvements (and larger effect sizes) in the parents and not in the burn survivors may relate to their pre-intervention mean scores, which were lower than the burn survivors’ baseline mean scores. After the intervention, the mean scores were similar. As indicated by several parents, and in line with the extant literature [45,46], guilt feelings and appraisals about their perceived responsibility for the burn event of their child may have affected their wellbeing. Self-compassion may facilitate looking at one’s own role from a more gentle perspective and may be an efficacious approach for parents of children with burns, particularly when they feel guilty. This is an important finding because previous research has shown that participants who scored high on self-compassion showed better psychological functioning [33]. Hawkins et al. (2019) associated higher levels of self-compassion with better coping patterns among parents of children recovering from acute burn injuries [12]. Examining whether parents with guilt feelings benefit from (early) interventions focused on increasing self-compassion may be an interesting avenue for future research.

It is worth noting that the ‘common humanity’ subscale element of self-compassion showed an increase in the parent group. The concept of common humanity refers to framing one’s suffering as part of being human and as an experience that connects the self to others rather than isolating oneself from them [33]. This increase in common humanity may be explained by the fact that the participants were practicing mindfulness in a group of peers where common distressful experiences were shared. For both groups, small effect sizes were noted regarding changes in the pre-, post-, and follow-up tests in depressive and PTSD symptom levels. This contrasts prior studies, for example, on parents of children with chronic conditions [42], but may relate to the low baseline levels of depressive and PTSD symptoms in the current study, leaving little room for improvement.

The current study has several limitations. First, the sample size was small, which may have affected the statistical power to detect significant changes. The effect sizes in the burn survivors were small to medium, which indicates that larger samples are needed. Replication in a larger study, preferably using a control group, is warranted. Second, the study used a convenience sample, which may have attracted highly motivated persons and people interested in mindfulness, and they may have more positive views of MBSR. Therefore, the results may not generalize to the burn population. Despite this limitation possibly causing sampling bias, this study adds that some aspects of MBSR, for example, relating to self-compassion, may apply to a larger group and warrant further research. Third, most participants experienced the burn event many years ago. The effects may have been larger if the intervention had been applied earlier after the burn event. Fourth, in this study, a spouse of a burn survivor participated in the study but did not fit specifically into one of the two groups. In the future, implementing an MBSR group for the significant others of burn survivors and studying the feasibility and effectiveness may be interesting. Fifth, one may consider other outcomes in future studies, such as quality of life and feelings of guilt, shame, and anger or more specific burn-related outcomes, such as anxiety appearance. Finally, the characteristics of the participants who were interested in the study but did not take part in the study were not registered. Therefore, it is unknown whether they differed from the participants.

## 5. Conclusions

This study contributes to the literature by providing preliminary evidence for the potential of an MBSR intervention to improve psychological well-being after a burn injury. Although the burn survivors’ effects were limited to a temporal improvement of their personal goals, for the parents, the picture was different, as there was a significant improvement regarding mindfulness (skills), self-compassion, and personal goals, including at follow-up. Future research needs to establish the effects of mindfulness on burn survivors and the parents of children with burns in a larger sample and with the inclusion of a control group. Pending this, this study provides preliminary evidence that the parents of children with burns may benefit from this group intervention.

## Figures and Tables

**Table 1:** MBSR program.

Session Theme	Mindfulness Practices/Exercises
1. The automatic pilot	Raisin exercise and the body scan
2. Attending and basic position	Attending versus interpreting, body scan and sitting meditation (breathing)
3. Boundaries and attending to the present	Meditation, seeing and hearing exercise, stretch out yoga
4. Stress and stress reactions	Sitting meditation, yoga and seeing-acting exercise
5. Coping with stress	Sitting and walking meditation (cognitions and feelings) and stress patterns
6. Cognitions are not facts	Sitting meditation and recognizing negative cognitions
7. Taking care of one’s self: Awareness for action	Sitting meditation, energy devourers, relapse prevention
8. Coping with stress and depressive moods in the future	Body scan, action plans, evaluation, and closing meditation

**Table 2:** Participants’ demographics and injury-related characteristics.

Characteristics	Burn Survivors ^1^ (*n = 8*)	Parents *(n = 9)*
Age, mean (*SD*)	49.4 (16.7)	52.2 (10.0)
Gender, female, *n* (%)	7 (88%)	6 (67%)
Education level, *n* (%) Primary Secondary Tertiary	0 (0%) 4 (50%) 4 (50%)	0 (0%) 6 (67%) 3 (33%)
Marital status, n (%) Single Married/partnered Divorced	1 (13%) 5 (62%) 2 (25%)	0 (0%) 9 (100%) 0 (0%)
Work status, *n* (%) Employed full-time Employed part-time Self-employed Retired Worker’s Compensation Act/Benefit	0 (0%) 5 (63%) 0 (0%) 2 (25%) 1 (12%)	1 (11%) 5 (56%) 2 (22%) 1 (11%) 0 (0%)
Years post-burn, Mdn (IQR)	16 (7-22)	5 (3–13)
TBSA burned, Mdn (IQR)	29 (9–52)	20 (6–63)
Face burned, *n* (%)	3 (38%)	6 (67%)
Genitalia burned, *n* (%)	1 (12%)	2 (22%)

^1^ One spouse was included in the burn survivor group. Results did not differ without the spouse.

**Table 3:** Participants’ satisfaction and credibility of the MBSR program.

Items	Burn Survivors Mean (SD)	Parents Mean (SD)
How useful did you find the intervention?	8.9 (1.5)	8.9 (0.9)
How satisfied are you with the intervention?	8.8 (1.2)	8.9 (0.9)
How confident are you that the intervention will help you cope better with the consequences of the burns?	8.4 (1.4)	8.6 (0.7)
How confident are you that the effects of the intervention will last more than 6 months?	7.6 (1.8)	8.7 (1.6)
Would you recommend the intervention to another person with burns or a parents of a child with burns?	9.3 (1.4)	9.4 (1.1)

**Table 4:** Participants’ ratings of agreeability of the MBSR program components.

	Not Pleasant		Neutral		Pleasant	
Items	Burn survivors *n* (%)	Parents *n* (%)	Burn survivors *n* (%)	Parents *n* (%)	Burn survivors *n* (%)	Parents *n* (%)
Listening to experiences of other participants	0	0	1 (12.5%)	0	7 (87.5%)	9 (100%)
Doing the training with peers	0	0	0	0	8 (100%)	9 (100%)
Meditation exercises	0	0	1 (12.5%)	1 (11%)	7 (87%)	8 (89%)
Yoga exercises	0	3 (33%)	3 (37.5%)	1 (11%)	5 (62.5%)	5 (56%)
Breathing exercises	0	0	1 (12.5%)	1 (11%)	7 (87.5%)	8 (89%)
Completing homework assignments	1 (12.5%)	0	6 (75%)	5 (56%)	1 (12.5%)	4 (44%)
Feelings and cognitions that arose as a result of practicing mindfulness	1 (12.5%)	1 (11%)	2 (25%)	1 (11%)	5 (62.5%)	(78%)
Contact with participants of the other mindfulness group (training day)	0	0	3 (37.5%)	1 (11%)	5 (62.5%)	8 (89%)

**Table 5:** Participants’ scores on the mindfulness subscales and the self-compassion subscale.

	Burn Survivors			Parents		
Subscales	Pre-Intervention Test M (SD)	Post-Intervention Test M (SD)	Follow-Up Test M (SD)	Pre-Intervention Test M (SD)	Post-Intervention Test M (SD)	Follow-Up Test M (SD)
**Mindfulness**						
Describing Effect size (d)	18.9 (2.2)	17.3 (3.1) 0.80	18.8 (4.7) 0.03	13.8 (2.9)	16.1 * (2.5) 1.50	16.9 * (4.9) 0.90
Awareness Effect size (d)	14.8 (3.5)	17.9 (2.3) 0.77	16.3 (2.8) 0.47	14.0 (4.7)	16.9 (4.8) 0.63	18.2 * (3.7) 1.06
Non-judgmental Inner experience Effect size (d)	16.0 (1.7)	17.6 (3.3) 0.47	16.8 (3.0) 0.24	17.5 (3.2)	16.3 (2.8) 0.27	16.9 (2.5) 0.18
Non-reactivity Effect size (d)	15.2 (2.9)	16.9 (2.9) 0.38	15.5 (2.5) 0.11	12.2 (2.5)	14.9 (3.0) 0.66	16.4 (4.9) 0.75
**Self-compassion**						
Self- kindness Effect size (d)	17.0 (4.7)	17.5 (5.0) 0.11	19.0 (5.2) 0.30	13.5 (4.9)	18.1 * (4.8) 1.37	18.0 (6.4) 0.57
Self-judgement Effect size (d)	17.4 (4.8)	18.8 (6.1) 0.21	18.1 (6.6) 0.13	15.0 (5.5)	16.1 (4.7) 0.33	18.9 *(6.3) 1.00
Common humanity Effect size (d)	15.1 (4.5)	14.8 (6.6) 0.06	13.9 (5.1) 0.27	13.3 (4.7)	19.1 *(4.5) 1.15	14.3 (3.5) 0.26
Isolation Effect size (d)	20.0 (5.2)	22.0 (4.3) 0.47	21.1 (5.1) 0.19	16.7 (4.5)	16.0 (4.3) 0.15	18.2 (5.9) 0.25
Mindfulness Effect size (d)	17.9 (5.8)	19.4 (4.1) 0.23	18.4 (4.9) 0.14	13.9 (3.7)	18.7 * (4.6) 1.26	18.2 * (5.0) 0.80
Over-identification Effect size (d)	18.6 (6.1)	21.5 (4.3) 0.45	20.3 (5.2) 0.28	17.4 (5.8)	16.8 (5.9) 0.14	20.3 * (5.7) 0.73

* Significant at 0.05 level; effect size indicated by d = Cohen’s d.

## Data Availability

The participants of this study did not give written informed consent for their data to be shared publicly, so due to the small sample and sensitive nature, the data are not available.
